# Supplementary material for: An open source three-mirror laser scanning holographic two-photon lithography system
Source: PLoS One. 2022 Apr 15;17(4):e0265678. doi: 10.1371/journal.pone.0265678 (PMC9012383; doi:10.1371/journal.pone.0265678)
Supplement: S2 File — (DOCX) [file pone.0265678.s002.docx]

| **Supplier** | **Part number** | **Description** | **Quantity** | **Block** | **Name in Figure 1** |
| --- | --- | --- | --- | --- | --- |
| Thorlabs | CEA1500 | Microscope body | 1 |  |  |
| Coherent | CHAMELEON VISION-S | fs-pulsed Tunable Ti:Sapphire Laser | 1 |  | Ti:Sapphire 690-1050nm <100fs |
| Thorlabs | CSA3000/M | Breadboard for microscope body 350mm x 275mm | 1 |  |  |
| Thorlabs | MB6060/M | Optical breadboard 600mm x 600mm | 1 |  |  |
| Thorlabs | C1515/M | Mounting post bracket | 4 |  |  |
| Thorlabs | PB4/M | Ø1.85" Studded Pedestal Base Adapter | 4 |  |  |
| Thorlabs | P350/M | Ø1.5" 350mm mounting post | 8 |  |  |
| Thorlabs | PF175B-P5 | Clamping Fork for Ø1.5" Pedestal Post, 5 Pack | 3 | Holography, Power control |  |
| Thorlabs | LC6W | 60mm Cage Cube | 1 |  |  |
| Thorlabs | ER4-P4 | Cage Assembly Rod, 4" Long, Ø6 mm, 4 Pack | 1 |  |  |
| Thorlabs | SM2CP2 | Externally SM2-Threaded End Cap | 1 |  |  |
| Thorlabs | LB1C/M | Cover Plate for 60 mm Cage Cube | 1 |  |  |
| Thorlabs | ZFM2020 | Motorized Module with 1" Travel | 1 |  |  |
| Thorlabs | CSN100 | Nosepiece for 1 Objective, M32 x 0.75 Threads | 1 |  |  |
| Thorlabs | M32M25S | Adapter External M32 x 0.75 and Internal M25 x 0.75 | 1 |  |  |
| Thorlabs | LB3C/M | Mounting Platform for 60 mm Cage Cube | 1 |  |  |
| Thorlabs | DMSP650L | Ø2" Shortpass Dichroic Mirror, 650 nm Cutoff | 1 |  | DM |
| Thorlabs | LB5C1 | Ø2" Optic Mount for 60 mm Cage Cube | 1 |  |  |
| Thorlabs | TR75/M | Ø12.7mm 75mm Post | 4 | Power control |  |
| Thorlabs | RA90RS/M | Right-Angle Ø25.0 mm to Ø1/2" Post Clamp | 5 | Imaging, Scanning, Holography |  |
| Thorlabs | RS150/M | Ø25mm 150mm mounting post | 3 | Imaging, Scanning |  |
| Thorlabs | POLARIS-CA25/M | Flexure Clamping Arm for Ø25 mm Posts | 7 | Imaging, Scanning, Holography |  |
| Physik Instrumente | P-725.11M | M25 x 0.75 Adapter (Microscope side) | 1 |  |  |
| Physik Instrumente | P-725.4CD | Piezo objective scanner | 1 |  |  |
| Physik Instrumente | P-725.11L | M25 x 0.75 Adapter (Objective side) | 1 |  |  |
| Olympus | XLPLN25XWMP2 | Objective lens | 1 |  | Lobj |
| Thorlabs | CSA1003 | Adapter Female D1N Dovetail and 60mm Cage System | 1 | Imaging |  |
| Thorlabs | WFA2002 | Epi-Illuminator Module for 1 Filters Cube | 1 | Imaging |  |
| Thorlabs | WFA4100 | 1X Camera Tube with C-Mount | 1 | Imaging | Limg |
| Hamamatsu | ORCA FUSION | Digital sCMOS camera | 1 | Imaging | sCMOS |
| Thorlabs | ER18 | Cage Assembly Rod, 18" Long, Ø6 mm | 4 | Imaging |  |
| Thorlabs | CP08/M | SM1-Threaded 30 mm Cage Plate with Flexure Clamping | 4 | Imaging |  |
| Thorlabs | SM1S10 | SM1 Lens Tube Spacer, 1" Long | 1 | Imaging |  |
| Thorlabs | M625L4 | 625 nm, 700 mW LED | 1 | Imaging | LED 625nm |
| Thorlabs | MDFM-MF2 | Microscopy Cube Assembly | 1 | Imaging |  |
| Thorlabs | BSW26R | 25mm x 36 mm 50:50 UVFS Plate Beamsplitter | 1 | Imaging | 50:50 NPBS |
| Semrock | FF01-512/630-25 | 512nm/630nm dual-band bandpass filter | 1 | Imaging | BPF |
| Semrock | FF01-680/SP-25 | 680nm blocking edge multiphoton short-pass emission filter | 1 | Imaging | NIR block |
| Thorlabs | AC254-030-A | f = 30mm, Ø1" Achromatic Doublet | 1 | Imaging | Lepi1 |
| Thorlabs | CP35/M | 30mm Cage Plate with Ø1" Double Bore | 1 | Imaging |  |
| Thorlabs | LBF254-040-A | N-BK7 Best Form Lens, Ø1", f = 40mm | 2 | Imaging | Lepi2, Lepi3 |
| Thorlabs | SM1RR | SM1 retaining ring | 3 | Imaging |  |
| Thorlabs | AC254-250-A | f = 250mm, Ø1" Achromatic Doublet | 1 | Imaging | Lepi4 |
| Thorlabs | TR50/M-P5 | Ø12.7mm 50mm Post, 5 Pack | 1 | Imaging |  |
| Thorlabs | RS300/M | Ø25mm 300mm mounting post | 2 | Imaging, Holography |  |
| Thorlabs | TL200-CLS2 | Laser Scanning Tube Lens, f = 200 mm | 1 | Scanning | Ltube |
| Thorlabs | LM2XY/M | Translating Lens Mount for Ø2" Optics | 2 | Scanning |  |
| Thorlabs | SL50-CLS2 | Laser Scanning Scan Lens, f = 50 mm | 1 | Scanning | Lscan |
| Thorlabs | SM2A11 | Adapter External SM2 and Internal SM30 | 1 | Scanning |  |
| Newport | 9065-X-M | Linear Stage, 14 mm Travel | 2 | Scanning, Power control |  |
| Newport | 9301 | Adjustment Screw, 12.7mm Travel | 2 | Scanning, Power control |  |
| Thorlabs | LCP08/M | 60 mm Cage Plate | 1 | Scanning |  |
| Thorlabs | ER12 | Cage Assembly Rod, 12" Long, Ø6 mm | 4 | Scanning |  |
| Thorlabs | GVS012/M | 2D Large 10 mm Diameter Beam Galvo System, Silver-Coated Mirrors | 1 | Scanning | 2D galvo XY scanner |
| Thorlabs | GVS011/M | 1D Large 10 mm Diameter Beam Galvo System, Silver-Coated Mirrors | 1 | Scanning | 1D galvo X scanner |
| Thorlabs | RS19/M | Ø25mm 19mm mounting post | 1 | Scanning |  |
| Thorlabs | RS25/M | Ø25mm 25mm mounting post | 3 | Scanning |  |
| Thorlabs | BA2F/M | Flexure Clamping Base / Post Mount, Ø25.0 mm | 2 | Scanning |  |
| Thorlabs | MBT616D/M | 3-Axis MicroBlock Compact Flexure Stage, Differential Micrometers | 3 | Scanning, Holography |  |
| Thorlabs | RB13P1/M | Adapter Plate for MBT616D/M | 3 | Scanning, Holography |  |
| Thorlabs | PF10-03-P01 | Ø1" Protected Silver Mirror | 7 | Scanning, Holography, Power control |  |
| Newport | SU100TW-F2K | Mirror Mount, ZeroDrift, 25.4mm | 5 | Scanning, Holography, Power control |  |
| Thorlabs | TR150/M | Ø12.7mm 150mm Post | 1 | Scanning |  |
| Thorlabs | BE1/M | Ø1.25" Studded Pedestal Base Adapter | 3 | Scanning, Power control |  |
| Thorlabs | PH50/M | Ø12.7mm 50mm Post Holder | 1 | Scanning |  |
| Thorlabs | CF125 | Clamping Fork, 1.24" Counterbored Slot | 3 | Scanning, Power control |  |
| Edmund Optics | #67-334 | Ø25mm f = 160mm, VIS-NIR Coated, Achromatic Lens | 1 | Holography | L4 |
| Thorlabs | SM1L03 | SM1 Lens Tube, 0.30" Thread Depth | 1 | Holography |  |
| Thorlabs | SM1ZM | SM1 Zoom Housing for Ø1" Optics | 1 | Holography |  |
| Thorlabs | ST1XY-S/M | XY Translator with 100 TPI Drives | 1 | Holography |  |
| Thorlabs | ER1.5-P4 | Cage Assembly Rod, 1.5" Long, Ø6 mm, 4 Pack | 1 | Holography |  |
| Thorlabs | CRM1LT/M | Cage Rotation Mount for Ø1" Optics | 1 | Holography |  |
| Newport | 10RP54-2B | Achromatic Waveplate, Quarter-Wave, 25.4mm Diameter | 1 | Holography | Quarter-wave plate |
| Thorlabs | RS100/M | Ø25mm 100mm mounting post | 5 | Holography, Power control |  |
| Thorlabs | RSH4/M | Ø25 mm 100mm Post Holder with Flexure Lock | 4 | Holography, Power control |  |
| Edmund Optics | #49-396 | Ø50mm f = 500mm, VIS-NIR Coated, Achromatic Lens | 1 | Holography | L3 |
| Newport | LP-2A-XYZ | Precision Lens Positioner, 2.0" | 2 | Holography |  |
| Thorlabs | RS75/M | Ø25mm 75mm mounting post | 7 | Holography, Power control |  |
| Thorlabs | RSH3/M | Ø25 mm 75mm Post Holder with Flexure Lock | 7 | Holography, Power control |  |
| Thorlabs | PF20-03-P01 | Ø2" Protected Silver Mirror | 2 | Holography |  |
| Newport | SN200-F2K | Mirror Mount, Suprema®, Clear Edge, 2.0" | 2 | Holography |  |
| Meadowlark Optics | HSP1920-500-1200-HSP8-785 | Spatial Light Modulator | 1 | Holography | SLM |
| Thorlabs | TR20/M | Ø12.7mm 20mm Post | 1 | Holography |  |
| Thorlabs | PH20/M | Ø12.7mm 20mm Post Holder | 1 | Holography |  |
| Thorlabs | RP01/M | Ø2" Manual Rotation Stage | 1 | Holography |  |
| Thorlabs | TR100/M | Ø12.7mm 100mm Post | 1 | Holography |  |
| Thorlabs | DT12/M | 1/2" Dovetail Translation Stage | 2 | Holography |  |
| Thorlabs | PH100/M | Ø12.7mm 100mm Post Holder | 1 | Holography |  |
| Edmund Optics | #49-391 | Ø50mm f = 150mm, VIS-NIR Coated, Achromatic Lens | 1 | Power control | L2 |
| Edmund Optics | #85-877 | Ø6,25mm f = -10mm, VIS-NIR Coated, Negative Achromatic Lens | 1 | Power control | L1 |
| Thorlabs | LMRA6.35 | Ø1/2" Adapter for Ø6.35 mm Optics | 1 | Power control |  |
| Thorlabs | LMR05/M | Lens Mount for Ø1/2" Optics | 1 | Power control |  |
| Thorlabs | PH75/M | Ø12.7mm 75mm Post Holder | 3 | Power control |  |
| Newport | 10RP52-2B | Achromatic Waveplate, Half-Wave, 25.4mm Diameter | 2 | Power control | Half-wave plate |
| Newport | RM25A | Optic Rotation Mount, 25.4mm | 2 | Power control |  |
| Newport | BSD-2R | Rail Periscope, 8.9 in. Height, 1 in. Mirrors | 1 | Power control |  |
| Newport | X26-512 | Optical Rail, Stainless Steel, 512 mm Length, 26 mm Width | 1 | Power control |  |
| Newport | CN26C | 90 Degree Rail Carrier, 50 mm Length | 1 | Power control |  |
| Conoptics | 350-80-02 | Electro Optical Modulator | 1 | Power control | Pockels cell |
| Thorlabs | SH05/M | Optical Beam Shutter | 1 | Power control | Shutter |
| Newport | 05FC16PB.5 | Cube Beamsplitter, Polarizing, 12.7mm | 1 | Power control | 50:50 PBS |
| Newport | UGP-KIT-1 | UGP Adapter Kit, 12.7mm Cube Riser | 1 | Power control |  |
| Newport | UGP-1 | Gimbal Prism Mount | 1 | Power control |  |
| Thorlabs | BT610/M | Beam trap | 1 | Power control | Beam dump |
| Thorlabs | PLS-XY | 2D Motorized Translation Stage | 1 |  |  |
| Thorlabs | MP150 | Rigid Stand with Platform | 1 |  |  |
| Thorlabs | KM100T | SM1-Threaded Kinematic Mount | 1 |  |  |
| Thorlabs | TR40/M | Ø12.7mm 40mm Post | 1 |  |  |
| Thorlabs | RA90/M | Right-Angle Clamp for Ø1/2" Posts | 1 |  |  |
| Thorlabs | UPH30/M | Ø12.7mm 30mm Universal Post Holder | 1 |  |  |
| Thorlabs | MCM3001 | Three-Channel Controller and Knob Box for 1" Travel Stages | 1 |  |  |
| Physik Instrumente | E-709 | Digital Piezo Controller | 1 |  |  |
| Thorlabs | LEDD1B | T-Cube LED Driver | 1 |  |  |
| Thorlabs | GPS011-EC | Galvo System Linear Power Supply | 2 |  |  |
| Thorlabs | KSC101 | K-Cube Shutter Controller | 1 |  |  |
