# Supplementary material for: An open source three-mirror laser scanning holographic two-photon lithography system
Source: PLoS One. 2022 Apr 15;17(4):e0265678. doi: 10.1371/journal.pone.0265678 (PMC9012383; doi:10.1371/journal.pone.0265678)
Supplement: Attachment — Submitted filename: ResponseToReviewers.docx [file pone.0265678.s005.docx]

**Editor**

Q1) Please ensure that your manuscript meets PLOS ONE's style requirements, including those for file naming. The PLOS ONE style templates can be found at 
<https://journals.plos.org/plosone/s/file?id=wjVg/PLOSOne_formatting_sample_main_body.pdf> and 
<https://journals.plos.org/plosone/s/file?id=ba62/PLOSOne_formatting_sample_title_authors_affiliations.pdf>

A1) We checked that the manuscript complies with the style requirements.

Q2) We note that the grant information you provided in the ‘Funding Information’ and ‘Financial Disclosure’ sections do not match.

When you resubmit, please ensure that you provide the correct grant numbers for the awards you received for your study in the ‘Funding Information’ section.

A2) We checked that all the funding sources are present in the “Funding Information” section.

Q3) Thank you for stating the following in the Competing Interests section:

"I have read the journal's policy and the authors of this manuscript have the following competing interests: M.D.V. and F.Pisanello are founders and hold private equity in OptogeniX srl, a company that develops, produces and sells technologies to deliver light into the brain. M.D.V.: OptogeniX srl (I). F.P.: OptogeniX srl (I)."

We note that you received funding from a commercial source: "OptogeniX srl"
Please provide an amended Competing Interests Statement that explicitly states this commercial funder, along with any other relevant declarations relating to employment, consultancy, patents, products in development, marketed products, etc.

Within this Competing Interests Statement, please confirm that this does not alter your adherence to all PLOS ONE policies on sharing data and materials by including the following statement: "This does not alter our adherence to PLOS ONE policies on sharing data and materials.” (as detailed online in our guide for authors <http://journals.plos.org/plosone/s/competing-interests>).  If there are restrictions on sharing of data and/or materials, please state these. Please note that we cannot proceed with consideration of your article until this information has been declared.

Please include your amended Competing Interests Statement within your cover letter. We will change the online submission form on your behalf.

A3) The new statement read as reported below. Please note that OptogeniX srl did not fund this research.

“I have read the journal's policy and the authors of this manuscript have the following competing interests: M.D.V. and F.Pisanello are founders and hold private equity in OptogeniX srl, a company that develops, produces and sells technologies to deliver light into the brain. This does not alter our adherence to PLOS ONE policies on sharing data and materials. OptogeniX did not fund the research described in this work. M.D.V.: OptogeniX srl (I). F.P.: OptogeniX srl (I).”

Q4) Please review your reference list to ensure that it is complete and correct. If you have cited papers that have been retracted, please include the rationale for doing so in the manuscript text, or remove these references and replace them with relevant current references. Any changes to the reference list should be mentioned in the rebuttal letter that accompanies your revised manuscript. If you need to cite a retracted article, indicate the article’s retracted status in the References list and also include a citation and full reference for the retraction notice.

A4) We checked the reference list.

**Reviewer 1**

Reviewer #1: The manuscript is relevant for the field of non-linear 3D lithography (laser 3D micro-/nano-printing) and its further developing. The results are original and useful as well as an opensource approach is attractive for the scientific and engineering community - it makes it easy to reproduce the revealed findings. Thus in principle the paper can be published in the selected Journal taking into account the indicated questions / remarks:

Q1) Abstract is not sufficiently specific. For instance the improved writing field by 2 is not compared to initial or the achieved value, nor the absolute value is given. As long as factor of 2 is not disruptive by itself, it should be clarified more technically (2 times corresponding to / in respect to what, or what was the achieved value?). In principle – 2 times is a reasonable improvement in the reported case context. Also, no comments on achieved resolution and throughput is provided, though they both are of critical importance while developing 2PP systems.

A1) We thank the reviewer for letting us improve the abstract. We now clearly state in the abstract the voxel size and the range of improvement of the WFOV from ~200µm to ~400µm, together with employed writing speed.

Q2) The obtained manufacturing speeds must be compared to the state-of-the-art achievements. As Authors provide projected (wide field) approach it should be compared to a single beam benchmarking reported results: Optics Express Vol. 27, Issue 11, pp. 15205-15221 (2019) •https://doi.org/10.1364/OE.27.015205. How many individual voxels per second does this novel proposed approach enables to print?

A2) According to the continuous line shown in Figure 4, written at 20 µm/s with a single continuous scan of three simultaneous spot, a rate of 2000 voxels/s was achieved considering the overlap between voxels. Compared with the reviewer’s reference there is a difference of one order of magnitude, that can be retrieved in part by increasing the number of spots projected simultaneously. We added these considerations in the discussion section.

“The system achieved a rate of 2000 voxels/s in writing 400 μm long lines (accounting for voxels overlap to obtain smooth structures), which is smaller than other value reported in literature [30]. This is in part due to the size of the galvanometric mirrors required to host the projected hologram, which reduce the maximum driving speed of the scanhead with respect to other systems [30]. The voxel rate can be partly improved by increasing the number of spots projected simultaneously. As the main aim of this study is exploiting the three mirror scanhead to enlarge the WFOV, this aspect will be investigated in future works.”

Q3) Figure 5 has strange units: 4000 nm, but 0.9 um? Why not 4 um and 900 nm?

A3) We edited the figure according to the reviewer’s comment.

Q4) Fig 4: continous => continuous.

A4) We corrected the typo.

Q5) 690 – 1050 nm < 100 fs laser. Have Authors performed the experiment by varying the wavelength? Seems like not, thus a specific set and applied central wavelength would give more useful information rather than the nominal parameter of the laser which might look misleading.

A5) We clarified further this aspect by removing the wavelength range and replacing it with 785 nm in Figure 1. We also specified in Section 2.1 that the wavelength used throughout the manuscript is 785 nm by adding the sentence

“All experiments reported in this manuscript were performed with the laser tuned to 785 nm”

Q6) The Authors mention “light intensity” as a parameter resulting to the polymerized shapes, which is indeed correct. Yet the fabrication parameters are provided in mW/um/s – a technical value, which is understandable and perhaps was convenient for the Authors to perform the carried research in the laboratory. However, in a non-linear light-matter interaction case (as is for 2-photon polymerization) it is the intensity (I, [W/cm^2]) the crucial parameter determining the type of interaction and modification of the material. See a recent prominent review paper discussing it: Nanophotonics, vol. 10, no. 4, 2021, pp. 1211-1242. <https://doi.org/10.1515/nanoph-2020-0551>. Namely Table 2 and Eq. 5 gives a comprehensive definition and explanation for it. So, in order this "open study" to make easier reproducable for other researchers - an intensity at the sample must be calculated. Power units like mW gives little information as different laser sources are used, and if holographic / projection lithography is applied as in the studied case, the multiple beams divide it by some factor (huge, perhaps like 10 or might be 100 - really interesting and important to know for choosing the right laser equipment and is a guide as a requirement for laser engineers). This can be estimated by the Authors and would be useful for the wide spectrum of readers.

A6) We thank the reviewer for letting us to be more accurate on this point. We added the estimation of the average intensity values in [TW/cm^2^] together with the technical values in mW/um/s. The reference suggested in this comment was added to the reference list.

Q7) No real 3D structure is shown! Why? This is the benchmark for the 2-photon polymerization technology – true 3D printing.

A7) We added a new Figure 6 showing examples of simple 3D structures, cited in the main test as in the following.

“Furthermore, as shown in Fig 6, the system was used to polymerize simple 3D structures in the shape of pyramids. As the memory buffer of the system is limited, the sample rate of the hardware/software interface was reduced to 32ksamples/s in this case.”

**Reviewer 2**

Reviewer #2: The Ms. reports an interesting development in the field of the optical setups for fast laser photopolymerization. It specifically tackles with the issue related to the relay between the two galvo mirrors needed for a xy scanning. It is well written and the conclusions are sound and well supported by data. I have only minor points:

Q1) in the ms. I cannot find a short description of how you get rid of the 0th order of the SLM. In the figure of the setup is marked. Are you adding a Fresnel lens hologram to do that? You can cite literature in this case, for example doi:10.3389/fncel.2014.00092 (2014).

A1) We thank the reviewer for noticing that the description of the 0^th^ order block is not clearly described. We added a sentence about it in section 2.2 of the manuscript, reading

“The unmodulated 0th order diffracted from the SLM was physically blocked with a razorblade placed in the focal plane of L3.”

Q2) in the introduction, on the citation of fabrication on hydrogels (page 2, ine 44), you can also cite other two very recent paper (doi:10.1002/adom.202000584) and review (doi:10.3390/s21175891)

A2) We added the references suggested by the reviewer.

Q3) Even if it is remarkable that you can increase the FOV with a real xy scanning system cooupled to a SLM, an alternative would be to use one galvo only and the SLM to parallelize the fabrication with a much simpler setup. Could you briefly comment about this possibility, if relevant?

A3) We thank the reviewer for letting us discuss this aspect. We added this consideration to the discussion section.

“A further simplification of the system would rely in the use of a single scanning mirror, avoiding the need of the compensating mirror, which could be particularly useful if patterning is needed preferentially along a single direction.”
